# Mothers’ Experiences of Respectful Maternity Care During Labor: A Descriptive Phenomenological Study at the KNUST Hospital

**DOI:** 10.1155/ogi/4552387

**Published:** 2026-07-30

**Authors:** Brandy Bonnah Swati, Adwoa Fosuaa, Gifty Dwomoh, Charllote Boateng, Veronica Millicent Dzomeku, Michael Arthur Ofori

**Affiliations:** ^1^ School of Public Health, University of Memphis, Memphis, USA, memphis.edu; ^2^ School of Nursing and Midwifery, Kwame Nkrumah University of Science and Technology, Kumasi, Ghana, knust.edu.gh; ^3^ Mathematical Sciences, Pan African University Institute for Basic Sciences, Technology and Innovation, Nairobi, Kenya, jkuat.ac.ke

**Keywords:** childbirth, health care, labor, labor care, maternity care, quality of care

## Abstract

Childbirth is a crucial event in a woman’s life, and the quality of care provided during this time can have a great impact on the mother’s well‐being. Unfortunately, disrespectful and abusive treatment during childbirth remains a concern in many healthcare settings, affecting women’s childbirth experiences and satisfaction with maternity care. This qualitative study employed a descriptive phenomenological approach to explore the lived experiences of mothers regarding respectful maternity care (RMC) during childbirth at a secondary‐level quasi‐government hospital in Kumasi, Ashanti region, Ghana. Individual, face‐to‐face interviews guided by a semistructured interview guide were conducted in English among 12 purposively sampled mothers who were seeking antenatal and postnatal services at the hospital. Interviews were transcribed verbatim and analyzed using inductive thematic analysis, aided by the NVivo software version 20.0. The study revealed that women perceived RMC as the absence of verbal abuse; consideration of women’s choices in delivery of care; showing concern for mother’s well‐being; and availability of knowledgeable healthcare providers. The study also revealed a range of experiences during childbirth. These included positive aspects such as friendly care and attention to mothers’ needs, as well as negative experiences, including mistreatment, lack of informed consent, and delay in receiving care. These experiences significantly influenced mothers’ overall satisfaction. It emphasizes the need for healthcare providers to receive training and sensitization on respectful communication and informed consent. Additionally, hospital management should work toward establishing feedback mechanisms to ensure the voices of women about the care they receive are heard.

## 1. Background

Labor is the process by which a woman’s body expels the fetus from the uterus, culminating in childbirth. While childbirth is a natural and often joyous event, it can also be a time of significant risk for both the mother and child [1]. Respectful maternity care (RMC) is now recognized as one of the most fundamental principles of global maternal health practice, reflecting a shift in perspective from clinical outcomes to women’s rights, dignity, and birthing experiences. While significant progress has been made in maternal health outcomes, research indicates that women have experienced persistent mistreatment during childbirth despite advances made within global maternal health frameworks. The WHO has indicated the need to include women’s experiences within the context of maternal health quality, as “women still endure mistreatment and disrespect during childbirth in healthcare settings.”

According to Hakimi et al. [2] “many women endure mistreatment and disrespect during childbirth in health facilities,” with global estimates for the prevalence of obstetric violence (OBV) reaching 59%, while the nonconsensual care being the most prevalent type. Disrespectful maternity care diminishes women’s sense of dignity, autonomy, and well‐being; in addition, mistreatment negatively impacts the maternal health status.

Besides addressing the problem in terms of prevalence, scholars argue that there are deeper social and structural factors associated with the issue. Sen et al. [3] state that the field suffers from “uncertain definitions, different language, and lack of agreed validated instruments.” At the same time, the authors argue that disrespect is influenced by existing inequalities and norms that exist within the system. It should be emphasized that “a request by a woman for attention, comfort, or pain relief in labor is not simply ignored; it is often ridiculed.”

Quality of care has two main dimensions—provision of care and user experience. The provision of care deals mainly with the quality of healthcare services rendered to patients. The user’s experience, on the other hand, encompasses the patient’s interactions with the care given [4]. Considering the link between these two dimensions, it is worth noting that a positive user experience is likely to influence the population to patronize healthcare services, when necessary. In contrast, a negative user experience may cause the population to lose trust in healthcare delivery [5]. This brings to light the significance of high‐quality care delivery during childbirth.

According to the World Health Organization [6], RMC refers to “the care organized for and provided to all women in a manner that maintains their dignity, privacy, and confidentiality, ensures freedom from harm and mistreatment, and enables informed choice and continuous support during labor and childbirth.” In hospitals all across the world, many women experience disrespectful and brutal treatment while giving birth [7]. In an attempt to prevent and eliminate disrespect and abuse during facility‐based childbirth, the World Health Organization [7] highlights the negative effects of this practice. Typical amongst them is the risk to life, health, and bodily integrity that it poses to these women.

In the past decade, numerous studies have brought attention to the mistreatment of women giving birth in low‐ and middle‐income countries, including physical and verbal abuse, age‐related discrimination (young or old mothers), and prejudice based on ethnicity or socioeconomic class. Other signs of abuse included giving care without permission, preventing a birth companion from being present, and depriving a woman of nourishment while she was in labor without her consent or a clinical indication [8–11]. In a study by Rosen et al. [12] in five sub‐Saharan African countries, many women have poor interactions with healthcare professionals and lack adequate information regarding their care. During the study, both verbal and physical abuse of women were reported, and in the open‐ended comments, desertion and neglect were the most often stated forms of disrespect and abuse [12].

RMC prioritizes women’s autonomy, dignity, and preferences, such as having birth companions, while ensuring the prevention of morbidity and mortality [13]. A study in Nigeria reported that midwives provided basic care, including confidentiality, food, and pain management, but failed in areas like avoiding physical abuse, maintaining privacy, and obtaining consent for interventions [14]. Abuse and disrespect during childbirth deter women from accessing postnatal services, undermining maternal health outcomes [4, 15]. Involving women and families as partners during childbirth promotes positive user experiences. Disrespectful care impedes progress in improving maternal health [10]. Monitoring RMC remains essential, but consensus on optimal metrics is lacking [16]. The causes of disrespect and abuse are complex and influenced by systemic dysfunctions and healthcare worker burnout [17]. Overburdened facilities with poor leadership and accountability further exacerbate these issues [10, 18, 19]. Addressing these challenges requires structural improvements and supportive environments for both patients and providers.

Maternal and newborn healthcare has improved significantly, with declining mortality rates due to better legislation, clinical practices, and professional care delivery [10, 20]. However, access to quality services remains uneven, especially in underdeveloped nations [13, 21]. Traumatic childbirth can lead to long‐term effects like poor mother–baby bonding, refusal to breastfeed, depression, and diminished quality of life, persisting for years in some cases [22, 23]. Abusive treatment during childbirth negatively impacts birth outcomes and leaves lasting memories. Disrespect and abuse also discourage women from returning to the same facility for future deliveries, compromising maternal healthcare utilization [4]. This issue is particularly prevalent in sub‐Saharan Africa, significantly affecting women’s experiences and perceptions of care [24–26]. Addressing this requires equitable access to high‐quality, respectful care worldwide.

Several studies on RMC in Ghana have focused on healthcare providers’ (HCPs’) experiences, knowledge, and perspectives [27, 28]. However, limited research has explored mothers’ experiences with RMC. Considering how important this concept is, it can be emphasized that mothers’ experiences during childbirth reflect the quality of care delivered by a health facility to childbearing women. Midwives’ understanding of RMC influences their ability to adhere to the protocols in providing care [27]. However, the result and/or effectiveness of the care can be determined by user experience. The experiences of mothers on RMC are therefore worth exploring.

The study sought to explore the lived experiences of women with care during labor by (i) assessing the perceptions of mothers on RMC, (ii) their experiences during childbirth care, and (iii) mothers’ satisfaction with childbirth care all at a secondary‐level quasi‐government hospital.

## 2. Materials and Methods

### 2.1. Study Design

The descriptive phenomenological methodology was employed to study women’s experiences of respect during childbirth. The open‐ended interview questions helped in exploring women’s experiences, perceptions, and feelings about the care that they received while giving birth. The study of a person’s actual experiences in the world is the main goal of this qualitative research method [29]. This design may be judged most effective for the current study since it acknowledges the subjective perception of the problem, and the many experiences participants have, and presents the findings in a form that directly reflects or nearly approaches the terminology used in the initial research question [30].

### 2.2. Study Settings and Population

The study was conducted at a secondary‐level quasi‐government hospital in Kumasi, Ashanti region. In 1952, the hospital was opened as a dressing station. Currently, the hospital serves approximately 200,000. It is the medical arm of a tertiary educational institution in Kumasi. The maternity unit of the hospital comprises an antenatal unit and a maternity ward. The maternity ward has a capacity of 13 beds, with a staff strength of 14 midwives. In addition to the staff, midwives are rotational nurses and midwives in varying numbers of at least 20 at a time, running different shifts. According to the unit records, there is an estimate of 100 deliveries per month. The population of the study included mothers who sought antenatal and postnatal services at the maternity unit of the hospital.

### 2.3. Sampling and Sample Size

A purposive sampling was adopted in recruiting participants for the study. A participant was considered as long as the inclusion and exclusion criteria were met as seen in the table below. The sample size was determined by data saturation, which is when no new information emerged from the interviews conducted. The saturation is not purely about the number of references but about the depth and richness of the data [31]. The inclusion criteria are as follows: (1) all mothers seeking antenatal and postnatal care at the maternity unit of the hospital and having had at least one delivery at the hospital and (2) mothers who could speak English or Twi, and mothers who were at least 18 years old. Exclusion criteria included (1) mothers unwilling to participate in the study, (2) a woman pregnant for the first time, and (3) mothers who could not speak or understand either English or Twi.

### 2.4. Data Collection and Statistical Analysis

A semistructured interview guide was drafted based on study objectives, and review of existing literature was utilized for the data collection in this study. Prior to the conduct of the study, pretesting of the instrument with similar subjects in a different facility was done to assess the reliability. Modifications were made based on the outcome of the pretest and expert comments. All participants who met the inclusion criteria were approached by the researchers during clinic days at the antenatal and postnatal clinics, and all aspects of the study were explained to them. Participants who consented to participate were recruited for the study and engaged in interviews, which were expected to last between 10 and 30 min per session. Participants provided written consent by signing or thumb‐printing a consent form before the interview sessions. There was one interviewer and an assistant who took field notes and reports. All interviews were audiotaped and transcribed later for analysis. Data collection began on September 25, 2023, to October 16, 2023.

All audiotaped interviews were transcribed verbatim in English and saved in separate Microsoft Word files. Transcribed data were read continuously to ensure that the exact statements from participants were captured in the transcript. The transcript was analyzed using Braun and Clarke’s [32] thematic analysis. Following transcription and proofreading, the transcribed interviews were read several times by two of the authors to gain a comprehensive understanding of the data. After reading through, initial codes were assigned to participants’ responses using NVivo version 20.0. The data were independently coded by the two authors, who subsequently met throughout the data analysis process to compare and contrast their codes until they came to a consensus on final codes and themes. All codes were solely derived from the data. The codes were then collated and grouped based on patterns and relationships between codes to form subthemes and, finally, main themes. Analysis of data was conducted using phenomenological techniques, where the researcher focused on participants’ accounts of lived experience before developing themes. After the development of the initial themes, the themes were reviewed and refined to ensure their coherence and relevance to the research objectives. The themes were revised, combined, separated, and discarded as necessary to accurately capture participants’ perspectives and experiences. Once the themes were reviewed and refined, they were defined by providing names.

### 2.5. Rigor/Trustworthiness of the Qualitative Study

The criterion of trustworthiness was discussed in four ways: credibility, dependability, confirmability, and transferability. The findings were presented with verbatim audio transcriptions, multiple readings of transcriptions for immersion in the data, and direct quotations from participants. The research assistant kept notes on the interviews conducted, which served as another data source for triangulating. Also, the use of a pretested semistructured interview guide, informed by the literature, and independent coding by two researchers using NVivo 20.0 enhanced dependability. The researchers then convened to discuss their code and come to an agreement, establishing an informal analytical trail of the analytical decisions taken.

Again, confirmability was encouraged by ensuring that all codes and themes were developed from the data, and not from a prior theory or framework. Throughout the data collection and analysis steps, the reflexivity process was continued in order to minimize the influence of researcher assumptions on the interpretation. Lastly, the extreme description of the study site, the participants, and procedures given in this discussion helps readers evaluate the transferability of the results. The sampling was purposive, with the aim of achieving data saturation, rather than breadth, to ensure depth and richness of data.

### 2.6. Ethics Approval and Consent to Participate

Ethical clearance was obtained from the Committee on Human Research, Publication, and Ethics (CHRPE) at the Kwame Nkrumah University of Science and Technology (KNUST) (CHRPE/AP/887/23). In addition, official approval was sought from the management of the hospital. All the participants were informed of their rights to voluntarily participate and withdraw from the study without any concerns. The purpose of the study was well explained to all participants. Hence, only participants who consented were included in the study. Participants were assured of confidentiality. Also, any data that can unveil participants’ identities were excluded from the transcripts to ensure participants’ anonymity.

## 3. Results

### 3.1. Demographic and Clinical Characteristics of Participants

In all, 12 women (12) within the age range of 29–38 years participated in the study. Most of the participants were working in the informal sector (*n* = 7) and had given birth once (*n* = 7). All of the women had been pregnant at least twice (Table 1).

**TABLE 1:** Demographic and clinical characteristics of participants.

Demographics	Frequency (*n*)
Age (range in years)	29–38
Occupation/employment	
Formal sector	3
Informal sector	7
Unemployed	2
Gravidity	
Primigravida	0
Multigravida	12
Parity	
Primipara	7
Multipara	5
Grand multipara	0

The transcribed interviews from the study were grouped and analyzed using inductive thematic analysis as shown in (Table 2). Three major themes and five subthemes were actively generated, which described mothers’ perceptions and experiences concerning their RMC and satisfaction with maternity care during labor.

**TABLE 2:** Themes and participant quotes.

Main theme	Participant quotes
*Subtheme*	
Perceptions of respectful maternity care	It’s the way nurses talk to patients and not to talk to them harshly (Ama).
	I understand respectful maternity care to be the relationship between the patient and the nurse on how to accept or disagree with the care given to you (Sena).
	It’s the way nurses treat patients and how knowledgeable they are (Mawuena).

Experiences of mothers during childbirth	
Friendly care	The midwives were friendly when I reported in labor; they always came around to know how I was doing, and they smiled, and were very polite. They treated me like a friend or neighbor (Akosua).
Caring for client needs	They were concerned about me; the midwives were all around me, encouraging me to push. The doctor too was nice. (Abena)
Mistreatment and disrespect	They are not practicing respectful maternal care. They sometimes shout at you and say things to you that make you look foolish, as if you don’t know anything (Akua).
Performance of procedures without consent	When I went to the labor ward, they gave me a bed and told me to open my legs for them to examine me, I didn’t like the fact that they didn’t seek my consent or explain to me before forcefully opening my legs to examine me. They don’t ask for your permission, which is very bad because it’s my body and they have to seek my consent before working on me (Akua).
Delay in receiving care	I reported to the hospital that day because I was not feeling well, and the doctor told me to do some laboratory tests. When I went to the lab, the lab technicians were not serious; they made me sit for a long time without attending to me, and that was frustrating. I then felt the edge to urinate, so I went to the washroom and unfortunately, I gave birth in the washroom. (Mina)

Satisfaction with care during labor	I am not satisfied. The authorities should sack those rude midwives.

### 3.2. Theme 1: Perceptions of RMC

Mothers described RMC as the relationship that exists between HCPs and the mother, the way care is delivered, and communication between HCPs and the mother. They perceived RMC as the absence of verbal abuse; consideration of women’s choices in delivery of care; showing concern for the mother’s well‐being, and availability of knowledgeable HCPs.

*It’s the way nurses talk to patients and not to talk to them harshly*. (Ama)

*I understand respectful maternity care to be the relationship between the patient and the nurse on how to accept or disagree with the care given to you*. (Sena)

*It’s the way nurses treat patients and how knowledgeable they are*. (Mawuena)

### 3.3. Theme 2: Experiences of Mothers During Childbirth

This theme describes the experiences of mothers during childbirth at the hospital, including positive and negative experiences. The experiences described were about the midwife–client relationship and delivery of care. Positive experiences included caring for the client’s needs and friendly care. Despite the positive experiences, mothers also spoke about negative experiences, including mistreatment and disrespect, and the performance of procedures without informed consent.

### 3.4. Friendly Care

Mothers were pleased with certain behaviors portrayed by midwives, such as respectful communication and cordial relationships with mothers. They described how midwives related to them like friends, spoke to them politely, and used good facial expressions. They reported that midwives were polite and did not mistreat them, even in situations when they expected such negative reactions.

*The midwives were friendly when I reported in labor; they always came around to know how I was doing, and they smiled and were very polite. They treated me like a friend or neighbor*. (Akosua)

*They didn’t say anything to me; they were polite and gentle. I thought they would shout at me for delivering in the washroom, but they didn’t*‐ (Wahiba)

### 3.5. Caring for Client Needs

Mothers shared experiences of how midwives and doctors showed concern and met their physical and emotional needs. They gave examples such as providing moral support during labor, having a personal midwife to care for them, and meeting physical needs such as providing water and massages. Mothers also spoke about how allowing their loved ones to be in the labor room helped them and made them feel loved.

*The doctors were very nice to me, as well also the midwives. When my water broke (membranes ruptured), they assigned one midwife to my room who monitored me till I delivered…she gave me water to drink when I needed it, and the doctor was there from time to time*. (Afua)

*They were concerned about me; the midwives were all around me encouraging me to push. The doctor too was nice*. (Abena)

*They massaged my back frequently and also allowed my husband to stay with me for a while, it helped me…When they allowed my husband to be with me, I felt loved*. (Afriyie)

### 3.6. Mistreatment and Disrespect

Verbal abuse in the form of insults and shouting at mothers during labor was reported by mothers. One mother who delivered in the car, because she was not well informed by midwives on the signs of labor, reported that midwives were angry and spoke to her badly. In such instances, mothers described that they felt “unwise” and “foolish”.

*They were very harsh, and if not because of my mother, I would have insulted them back…I didn’t like the way they spoke to me, especially one particular midwife. They made me look unwise, but it wasn’t my fault that the midwives at the antenatal didn’t educate me on the signs of labor*. (Samira)

*They are not practicing respectful maternal care…they sometimes shout at you and say things to you that make you look foolish, as if you don’t know anything*. (Akua).

### 3.7. Performance of Procedures Without Consent

Bodily autonomy and the need for midwives to respect this right were mentioned by mothers. Mothers expressed their dissatisfaction with the failure of midwives to provide information and seek their consent before performing procedures on them.

*When I went to the labor ward, they gave me a bed and told me to open my legs for them to examine me. I didn’t like the fact that they didn’t seek my consent or explain to me before forcefully opening my legs to examine me. They don’t ask for your permission, which is very bad because it’s my body, and they have to seek my consent before working on me*. (Akua)

### 3.8. Delay in Receiving Care

This theme is related to delays in receiving care at other units of the hospital, other than the labor ward. One mother described how the delay in receiving care at the lab contributed to her delivery in the washroom.

*I reported to the hospital that day because I was not feeling well, and the doctor told me to do some laboratory tests. When I went to the lab, the lab technicians were not serious; they made me sit for a long time without attending to me, and that was frustrating. I then felt the urge to urinate, so I went to the washroom and unfortunately, I gave birth in the washroom*. (Mina)

### 3.9. Theme 3: Satisfaction With Care During Labor

Satisfaction with care during labor emerged from participants’ subjective appraisals of their overall care experiences. Consistent with qualitative approaches to maternity care research, satisfaction was understood as an experiential phenomenon directly shaped by encounters with respectful or disrespectful treatment [33]. Mothers who reported positive interactions with midwives expressed satisfaction, while those who experienced mistreatment, inadequate information, or delays expressed dissatisfaction.

Mothers’ satisfaction with care received during labor was determined by their experiences—those who felt they were treated well said they were satisfied with the care provided, while those who had negative experiences were not pleased. Those who expressed dissatisfaction with the care provided felt RMC was not practiced because some midwives were rude and others talked about inadequate provision of education.

*Information given to patients is not enough. I was not given much education during my previous pregnancy, and I believe that’s why I lost them…Am not satisfied with my care, but what can you do?*

*I am not satisfied. The authorities should sack those rude midwives*.

Mothers attributed the problem of inadequate education to the increased workload on midwives.

*They are always busy because a lot of people come for antenatal and the only education we get there is the one they show on TV, and not all of us understand English*.

Dissatisfaction among participants was multidimensional, rooted in interpersonal rudeness, inadequate provision of health education, and structural constraints such as midwife workload. These findings suggest that satisfaction with maternity care is shaped not only by individual interactions but also by systemic factors that influence the quality of care delivered [8].

## 4. Discussion

### 4.1. Perceptions on RMC

Women’s perceptions of RMC focused on the interpersonal skills of HCPs more than their technical skills. They emphasized the importance of the relationship and communication between HCPs and mothers. Mothers expected HCPs to refrain from harsh or disrespectful communication. They viewed the absence of harsh words as an indicator of respectful communication. Thus, their responses indicate that how care is delivered is as important as the care itself. The perception of women in this study agrees with the RMC domain of “being free from harm and mistreatment” as defined by Shakibazadeh et al. [33] and is similar to the findings of Esan et al. [34] in a qualitative study conducted in Nigeria.

RMC emphasizes respecting women’s choices and strengthening their capabilities during childbirth. Women in this study highlighted the importance of their choices being considered in care delivery, contrasting findings from Esan et al. [34], where women deferred decision‐making entirely to HCPs. Respecting women’s choices aligns with the principles of autonomy and shared decision‐making, requiring HCPs to seek and respect mothers’ preferences on issues like birth positions, mobility, and nutrition during labor. When women’s preferences differ from medical recommendations, HCPs should provide clear explanations to enable informed decisions, empowering women in their care. Additionally, women valued knowledgeable and competent HCPs, reinforcing the importance of skilled and motivated staff as a core aspect of RMC. This dual focus on shared decision‐making and professional competence ensures high‐quality RMC.

### 4.2. Experiences of Mothers During Childbirth

The theme of “experiences of mothers during childbirth” highlights both positive and negative hospital experiences. Positive experiences included friendly attitudes, respectful communication, and attention to mothers’ needs, similar to findings among Palestinian, Iraqi, and Syrian refugee women in Lebanon [35]. Mothers valued empathy and good interpersonal skills from midwives and doctors, showing how respectful treatment enhances childbirth experiences. Emotional well‐being was further supported by having birth companions, who provided comfort, reduced mistreatment, and assisted with pain management through physical and emotional support. The presence of companions has been linked to shorter labor, fewer cesarean sections, higher satisfaction with care, and early breastfeeding initiation, underscoring their critical role in improving maternity outcomes.

Some women reported negative childbirth experiences, including mistreatment, lack of consent for procedures, and delays in care. In Ghana, studies show that mothers often face abuse during childbirth, with some midwives believing that verbal and physical abuse can prevent complications [27]. Verbal abuse left mothers feeling disrespected and devalued, negatively impacting their emotional well‐being and overall experience. Mothers also criticized the failure to obtain informed consent, viewing it as a violation of their bodily autonomy, aligning with findings from Sweden where women valued involvement in decision‐making. However, Esan et al. [34] found that some women trusted HCPs entirely and did not consider consent necessary. This highlights a power imbalance between HCPs and patients, especially in low‐resource settings, where women may feel unable to question authority or seek information, perpetuating the disregard for informed consent and autonomy.

### 4.3. Satisfaction With Care During Labor

RMC and women’s experiences of satisfaction were studied together since women’s perception of respect could affect their experiences of childbirth satisfaction. Satisfaction with care received during labor was primarily determined by the nature of the mother’s experience. Thus, the kind of care received during labor and how it is delivered significantly impact the overall childbirth experience and satisfaction with care. One of the reasons for dissatisfaction with care was the inadequate provision of education during the antenatal period. Mothers felt that inadequate education led to negative neonatal outcomes, such as neonatal morbidity. The role of education in preserving and improving maternal and neonatal health outcomes during the perinatal period cannot be underestimated. Mothers recognized that this problem of inadequate provision of education was due to the limited time, which resulted from the large number of pregnant women midwives had to attend to. Hospital management can curb this problem by employing more midwives to cater for the increasing maternal population. This may help reduce the workload on midwives and spare them more time to provide the education needed and counsel to mothers.

### 4.4. Limitations

There are some limitations of this study. First, the study took place in a secondary‐level hospital in Kumasi. This limits the applicability of its results to other types of hospitals, regions, or health systems. Second, while appropriate for the purpose, there is possible selection bias with purposive sampling. Third, the small sample size limits the possibility for transferability, consistent with the principles of qualitative inquiry. Fourth, the language‐based inclusion criteria (English or Twi) could have missed some women who spoke different regional languages, which may have missed some perspectives. Finally, purposive sampling of the socioeconomic background, ethnicity, and maternal age has not been conducted. These gaps could be filled with future studies using larger, stratified, and multisite sample collections.

## 5. Conclusion

RMC is essential for ensuring safe and positive childbirth experiences for women. The findings of this study highlight the importance of respectful communication, empathy, friendly care, and informed consent in enhancing the quality of care during labor. Improvement strategies for RMC should include training HCPs and permitting birth companions. Hospitals need to place high value on informed consent and comprehensive patient education and teaching women about reproductive health is empowering women. Despite the use of purposive sampling, socioeconomic background, ethnicity, and the age of mothers have not been used to stratify the population under study. All the above factors are likely to affect women’s experience of respectful care and should therefore be considered in future studies.

## Author Contributions

All authors contributed immensely to the development of this work.

## Funding

There has not been any financial support for this work that could have influenced its outcome.

## Disclosure

The abstract of this manuscript was presented at the TPHA and APHA 2025 Annual Meeting and Expo [36].

## Conflicts of Interest

The authors declare no conflicts of interest.

## Data Availability

Data are available from the corresponding author upon request.
